# Gelam Honey Protects against Gamma-Irradiation Damage to Antioxidant Enzymes in Human Diploid Fibroblasts

**DOI:** 10.3390/molecules18022200

**Published:** 2013-02-11

**Authors:** Tengku Ahbrizal Farizal Tengku Ahmad, Zakiah Jubri, Nor Fadilah Rajab, Khairuddin Abdul Rahim, Yasmin Anum Mohd Yusof, Suzana Makpol

**Affiliations:** 1 Department of Biochemistry, Faculty of Medicine, Universiti Kebangsaan Malaysia, Jalan Raja Muda Abdul Aziz, Kuala Lumpur 50300, Malaysia; 2 Division of Agrotechnology and Biosciences, Malaysian Nuclear Agency, Bangi, Kajang 43000, Malaysia; 3 Department of Biomedical Sciences, Faculty of Health Sciences, Universiti Kebangsaan Malaysia, Jalan Raja Muda Abdul Aziz, Kuala Lumpur 50300, Malaysia

**Keywords:** Gelam honey, gamma-irradiation damage, antioxidant enzymes, human diploid fibroblasts

## Abstract

The present study was designed to determine the radioprotective effects of Malaysian Gelam honey on gene expression and enzyme activity of superoxide dismutase (SOD), catalase (CAT) and glutathione peroxidase (GPx) of human diploid fibroblasts (HDFs) subjected to gamma-irradiation. Six groups of HDFs were studied: untreated control, irradiated HDFs, Gelam honey-treated HDFs and HDF treated with Gelam honey pre-, during- and post-irradiation. HDFs were treated with 6 mg/mL of sterilized Gelam honey (w/v) for 24 h and exposed to 1 Gray (Gy) of gamma rays at the dose rate of 0.25 Gy/min. Gamma-irradiation was shown to down-regulate *SOD1*, *SOD2*, *CAT* and *GPx1* gene expressions (*p* < 0.05). Conversely, HDFs treated with Gelam honey alone showed up-regulation of all genes studied. Similarly, SOD, CAT and GPx enzyme activities in HDFs decreased with gamma-irradiation and increased when cells were treated with Gelam honey (*p* < 0.05). Furthermore, of the three different stages of study treatment, pre-treatment with Gelam honey caused up-regulation of *SOD1*, *SOD2* and *CAT* genes expression and increased the activity of SOD and CAT. As a conclusion, Gelam honey modulates the expression of antioxidant enzymes at gene and protein levels in irradiated HDFs indicating its potential as a radioprotectant agent.

## 1. Introduction

Ionizing irradiation is known to induce oxidative stress due to overproduction of reactive oxygen species (ROS) causing disturbances to cellular metabolism. Radioactivity exists either naturally on Earth or from cosmic rays or is synthetically produced at radiation facilities such as nuclear reactor plants, by X-rays, CT scans and radiotherapy [1,2]. It is estimated that 80% of radiation exposure was received from natural sources [3]. Although the exposure from artificial sources was low as compared to exposure from natural sources, the increase in radiation applications has resulted in increased numbers of humans exposed to radiation. Currently there are about 434 commercial nuclear power reactors operating in 31 countries, 60 reactors under construction and some 150 reactors are firmly planned [4]. Although the level of radiation exposure for radiation workers is monitored, they are more frequently exposed to radiation as compared to the public and may have higher risk to face radiation accidents. For patients who undergo radiotherapy treatment, the radiation does not only kill the cancer cells but also produced side effects whereby the irradiated cancer cells will directly transmit damaging signals to normal non-irradiated cells and produce similar effects as in the irradiated cancer cells [5].

Ionizing radiation consists of energetic particles which can penetrate living tissues or cells. It decomposes water molecules in the cells and produces H_2_O^+^ ion resulted in the production of several radicals such as hydroxyl ion (OH^−^), superoxide anion (O_2_^−^), hydrogen ion (H^−^) and electron (e^−^) [6,7]. These free radicals will then attack nucleic acids, lipid membranes and proteins to produce secondary radical, hydrogen peroxide (H_2_O_2_). Hydrogen peroxide can be converted into hydroxyl ion through Fenton reaction [8,9,10]. High level of free radicals existence causes toxicity to the cells and induces mutation and carcinogenesis, while low level of free radicals leads to cell proliferation inhibition and apoptosis induction [11].

Amongst the different organs in our body, the skin is the largest and the first organ to be exposed to external ionizing radiation [12]. Skin consists of mixed types of cells such as keratinocytes, melanocytes, Langerhans cells, Merkel’s cells, nerve and glandular cells and fibroblasts. Fibroblasts are the most common cells found in loose connective tissue which are responsible for producing tissue elements or their precursors. Fibroblasts have been used as control cells in studies involving other cell types and in pharmacological tests as well as in skin reconstruction [13]. Due to its sensitivity to radiation, fibroblasts have been used as a baseline in a study elucidating the biological effects of therapeutic irradiation [14]. Previous study has shown that early stages of radiation exposure induces DNA damage in human diploid fibroblasts (HDFs) [15] whereas late effects of radiation induces post-mitotic damage, which later induces senescent phenotype, activates cascades of profibrotic chemokines and cytokines. These damages lead to skin and pulmonary fibrosis as shown in animal models and clinical studies [16].

Cells are naturally equipped with antioxidant defense systems to counterbalance free radical production and protect against oxidative stress via an interacting network of antioxidant enzymes. There are three primary antioxidant enzymes involved in detoxifying free radicals, *viz*. superoxide dismutase (SOD), catalase (CAT) and glutathione peroxidase (GPx). Superoxide dismutase (SOD) is the first enzyme involved in antioxidant defense mechanisms. Two common SOD isoforms, CuZn-SOD (SOD1) and MnSOD (SOD2) are localized in intracellular cytoplasm and mitochondrial matrix, respectively [17]. SOD decomposes O_2_^−^ ion to O_2_ and H_2_O_2_ molecules [11]. Subsequently, CAT and GPx decomposes H_2_O_2_ to O_2_ and H_2_O molecules. CAT is located in the peroxisomes and cytosol whereas glutathione peroxidase (GPx) is present in the mitochondrial matrix of the cell [7,18].

Although aerobic organisms possess an antioxidant defense system, damage occurs when free radical production exceeds the limit of the system’s ability. Radiation was found to affect the activity of antioxidant enzymes [19,20,21]. Previous studies have shown that ionizing radiation increased SOD activity and decreased catalase and glutathione reductase activities in fibroblast cells isolated from mice [20]. The increase in SOD activity indicated higher decomposition of O_2_^−^ ion to H_2_O_2_ in the cells while decreased CAT and GPx activities showed less H_2_O_2_ was being reduced to O_2_ and H_2_O molecules. This condition indirectly results in accumulation of H_2_O_2_ in the cells. In order to prevent or reduce the radiation damage caused by free radicals and to identify a potential radioprotectant agent, several studies have been performed. Animal studies have shown that selenium and vitamin E increased antioxidant enzyme levels in normal cells [22]. Singh *et al*. showed that α-tocopherol succinate increased the survival rate of mice upon gamma-irradiation [23]. Melatonin has also been shown to reduce oxygen radicals in mitochondria when exposed to radiation by increasing GPx regeneration [24].

Another natural compound which has the potential as a radioprotectant agent is honey. Clinical studies done by Biswal *et al*. indicated that Tualang honey decreased mucositis production in patients with head and neck cancer who underwent radiotherapy treatment [25]. Another type of Malaysian honey which is Gelam honey was reported to contain several antioxidant active compounds such as quercitin, caffeic acid, chlorogenic acid [26], gallic acid, ferulic acid, benzoic acid and cinnamic acid [27]. Gelam honey has been shown to protect against oxidative stress in young and middle aged rats by modulating antioxidant enzyme activities [28]. The effects of Gelam honey against radiation damage however have not been reported. Therefore this study aimed to elucidate the protective effects of Gelam honey against radiation damage by determining the gene expression and enzyme activities of SOD, CAT and GPx in HDFs upon exposure to 1 Gray (Gy) of gamma-rays.

## 2. Results

### 2.1. SOD1, SOD2, CAT and GPx1 Gene Expression

**Figure 1:**
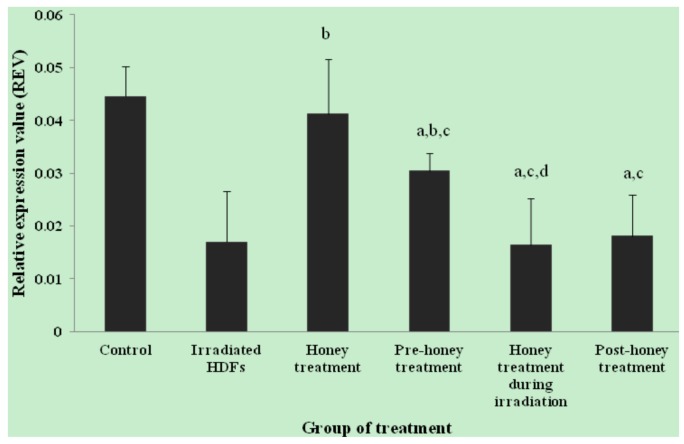
Expression of *SOD1* gene in different HDFs treatment groups. HDFs were pre-, during- and post-treated with 6 mg/mL of Gelam honey for 24 h and exposed to 1 Gy of gamma rays. Results are expressed as means + S.D (n = 6). ^a^ Denotes *p* < 0.05 compared to untreated control, ^b^ *p* < 0.05 compared to irradiated HDFs, ^c^ *p* < 0.05 compared honey treated-HDFs, ^d^ *p* < 0.05 compared to pre-honey treated-HDFs.

**Figure 2:**
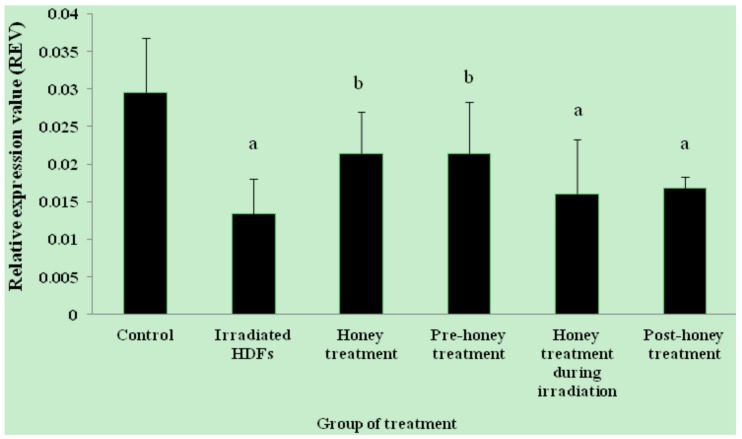
Expression of *SOD2* gene in different HDFs treatment groups. HDFs were pre-, during- and post-treated with 6 mg/mL of Gelam honey for 24 h and exposed to 1 Gy of gamma rays. Results are expressed as means + S.D (n = 6). ^a^ Denotes *p* < 0.05 compared to untreated control, ^b^ *p* < 0.05 compared to irradiated HDFs.

**Figure 3:**
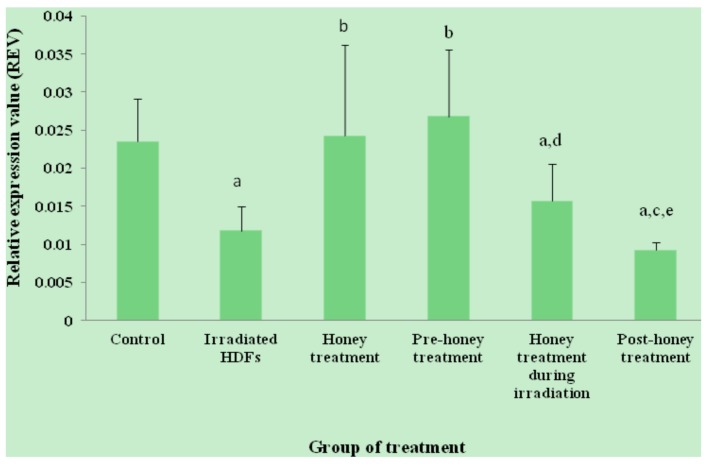
Expression of *CAT* gene in different HDFs treatment groups. HDFs were pre-, during- and post-treated with 6 mg/mL of Gelam honey for 24 h and exposed to 1 Gy of gamma rays. Results are expressed as means + S.D (n = 6). ^a^ Denotes *p* < 0.05 compared to untreated control, ^b^ *p* < 0.05 compared to irradiated HDFs, ^c^ *p* < 0.05 compared honey treated-HDFs, ^d^ *p* <0.05 compared to pre-honey treated-HDFs, ^e^ *p* < 0.05 compared to honey treated-HDFs during irradiation.

**Figure 4:**
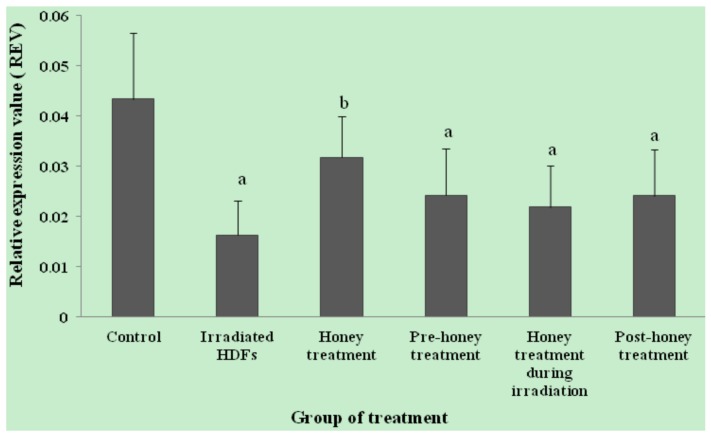
Expression of *GPx1* gene in different HDFs treatment groups. HDFs were pre-, during- and post-treated with 6 mg/mL of Gelam honey for 24 h and exposed to 1 Gy of gamma rays. Results are expressed as means + S.D (n = 6). ^a^ Denotes *p* < 0.05 compared to untreated control, ^b^ *p* < 0.05 compared to irradiated HDFs.

The relative expression value (REV) of *SOD1* gene was significantly decreased in HDFs exposed to 1 Gy of gamma-rays as compared to untreated control (*p* < 0.05) (Figure 1). In contrast, *SOD1* gene was up-regulated in honey-treated group as compared to irradiated HDFs. Among the three different treatments, pre-treatment with Gelam honey before irradiation caused up-regulation of *SOD1* gene as compared to irradiated HDFs without Gelam honey treatment. Similar decrease in *SOD2* gene expression was observed in irradiated HDFs (*p* < 0.05) as compared to untreated control (Figure 2). *SOD2* gene was up-regulated in honey-treated HDFs and irradiated HDFs with pre-treatment of Gelam honey as compared to irradiated HDFs without honey treatment. Irradiated HDFs also showed down-regulation of *CAT* gene expression as compared to untreated control (Figure 3). *CAT* gene expression was significantly up-regulated in honey-treated HDFs and irradiated HDFs with pre-treatment of honey as compared to irradiated HDFs group. Similarly, *GPx1* gene was significantly down-regulated in irradiated HDFs as compared to untreated control. Only honey-treated HDFs showed significant up-regulation of *GPx1* gene expression as compared to irradiated HDFs without honey treatment (Figure 4).

### 2.2. Antioxidant Enzyme Activity

The antioxidant enzyme activities were determined by measuring the specific activity of SOD, CAT and GPx (Table 1). SOD activity decreased in irradiated HDFs as compared to untreated control. However SOD activity was increased in honey-treated HDFs, irradiated HDFs pre-treated with Gelam honey and HDFs treated with Gelam honey during irradiation. Similar results were observed in CAT activity. Gamma-irradiation decreased CAT activity in irradiated HDFs as compared to untreated control HDFs. CAT activity was significantly increased in honey-treated HDFs and irradiated HDFs pre-treated with Gelam honey as compared to irradiated HDFs group. GPx activity was significantly decreased in irradiated HDFs as compared to untreated control group. Only Gelam honey-treated HDFs showed significant increase in GPx activity as compared to irradiated HDFs.

## 3. Discussion

The effort to develop radioprotectant agents has been initiated decades ago in order to protect human cells from radiation damage whereby synthetic and natural compounds have been studied [22,23,24]. In this study, the effectiveness of Malaysian Gelam honey treatment in protecting HDFs against gamma-irradiation was evaluated. Gelam honey at the concentration of 6 mg/mL was selected as our previous cytotoxicity study indicated that this concentration maintained HDFs viability at 86.7% with free radical scavenging activity of 0.7% and total antioxidant power of 16.3 μM [15]. Although Gelam honey at concentration of 1 mg/mL maintained HDFs viability at 92.2%, this concentration was not chosen for the study because its free radical scavenging activity and total antioxidant power were less than 0.46% and 5 μM, respectively. On the other hand, Gelam honey at the concentration of 10 mg/mL exhibited 12.2% of free radical scavenging activity and 22.0 μM of total antioxidant power of Gelam honey, but the HDFs viability was low (70.0%). Therefore, the concentration of 6 mg/mL was chosen as it maintained cells viability with optimum antioxidant activities [15].

In this study, the role of antioxidant enzymes in detoxifying free radicals generated from radiation exposure was evaluated by determining the expression of *SOD1*, *SOD2*, *CAT* and *GPx1* genes as well as the SOD, CAT and GPx enzyme specific activities. ROS produced from radiation exposure was shown to indirectly attacked antioxidant enzymes and affects the functions of these enzymes [19]. Our results showed that SOD, CAT and GPx activities decreased with gamma-irradiation. Similar results by Davis *et al*. indicated that gamma-irradiation decreased catalase and glutathione reductase activities in irradiated fibroblasts isolated from mice [20]. Kang *et al*. reported that radiation decreased SOD and CAT activities in Chinese hamster lung fibroblasts (V79-4) which were exposed to gamma-rays [21]. Decreased SOD activity resulted in decreased reduction of superoxide ion to H_2_O_2_ and O_2_ in the cells. Okunieff *et al*. reported that ionizing radiation can damage mitochondrial function and accumulation of ROS in the cells can cause further damage to the mitochondria [24]. This may explains the decreased in *SOD2* gene expression in HDFs as this enzyme is located in the mitochondria.

Different starting times for cell treatment have also been studied in order to determine the most suitable time to treat HDFs upon exposure to radiation [15]. In this study, HDFs were treated with Gelam honey before, during and post-irradiation to ensure the antioxidant enzyme activities were well protected from radiation damage. Among the three different treatment times studied, pre-treatment with Gelam honey before HDFs were exposed to radiation resulted in up-regulation of SOD activity as compared to irradiated HDFs without Gelam honey treatment. Subsequently, CAT activity was also increased indicating that H_2_O_2_ was decomposed to O_2_ and H_2_O molecules. This finding revealed that pre-treatment with Gelam honey did protect antioxidant enzymes against radiation damage.

Similar results however were not observed when HDFs were treated with Gelam honey during irradiation. Although both *SOD1* and *SOD2* genes were not up-regulated when HDFs were treated with Gelam honey during irradiation, the SOD specific activity was increased. This could be due to the presence of SOD3. Previous study reported that besides SOD1 and SOD2, extracellular CuZnSOD (EC-SOD, SOD3) is another isoform of SOD [17,18] which was not analysed in this study. Therefore there is a possibility that the increase in SOD specific activity observed in HDFs treated with Gelam honey during irradiation was due to the interaction between SOD3 and Gelam honey. In contrast, neither CAT nor GPx activity was increased in HDFs treated with Gelam honey during irradiation. Previous reports stated that honey contains H_2_O_2_ produced from the reaction catalysed by activated glucose oxidase which oxidised glucose molecule to form gluconic acid and H_2_O_2_ [29,30]. This may lead to the accumulation of H_2_O_2_ which exceeds the limit existence in the cells, which subsequently destroys the functions of CAT and GPx enzymes during irradiation.

Meanwhile, Gelam honey treatment after irradiation did not produce any significant protective effects against radiation damage on the SOD, CAT or GPx specific activities of HDFs. A study done by Davis *et al*. indicated that CAT activity did not increase when mice fibroblast skin cells were treated with 50 μM α-lipoic acid after being exposed to 2 Gy of gamma-rays [20]. However, CAT activity was significantly increased in cells treated with 100 μM α-lipoic acid after radiation exposure. Our previous findings showed that Gelam honey did not protect against DNA damage or increase cell survival rate when HDFs were treated during- and post-irradiation while the same dosage of honey showed protection when cells were treated before irradiation [15]. This observation indicates that the protection against radiation damage is very much affected by the treatment starting time.

## 4. Experimental

### 4.1. Sterilization of Gelam Honey

Malaysian monofloral Gelam honey is produced by *Apis mellifera*, and the major nectar and pollen collected by the bees is from the plant *Melaleuca cajuputi* Powell, which is known locally as the “Gelam tree”. It was purchased from the Department of Agriculture, Batu Pahat, Johor, Malaysia. Gelam honey was packed in tight cap plastic bottles and placed in a box before sending to SINAGAMA, Malaysian Nuclear Agency. The sterilization process was carried out using cobalt-60 source (Model JS10000, Atomic Energy of Canada Ltd, Ontario, Canada). The box which contained Gelam honey was carried into a gamma-radiation chamber and circled the cobalt-60 source for 5 times to reach the dose of 25 kiloGray (kGy). The dose was automatically calculated by cobalt-60 machine. The irradiated Gelam honey was then kept in the dark at room temperature.

### 4.2. Cell Culture Protocol

Primary HDFs were derived from foreskins of three 9 to 12 year-old boys after circumcision. Written informed consents were obtained from parents of all subjects. The samples were aseptically collected and washed several times with 75% alcohol and phosphate buffered saline (PBS) containing 1% antibiotic-antimycotic solution (PAA, Pasching, Austria). After removing the epidermis, the pure dermis was cut into small pieces and transferred into a falcon tube containing 0.03% collagenase type I solution (Worthington Biochemical Corporation, Lakewood, NJ, USA). The pure dermis was digested in an incubator shaker at 37 °C for 6–12 h. Cells were then rinsed with PBS before being cultured in Dulbecco Modified Eagle Medium (DMEM, Flowlab™, North Ryde, Australia), 10% fetal bovine serum (PAA), 10,000 μg/mL penicillin/streptomycin (Gibco, Grand Island, NY, USA), 250 μg/mL amphotericin B (PAA), 100 mg/mL gentamycin (PAA) and incubated in 5% CO_2_ atmosphere at 37 °C. This research has been approved by the National University of Malaysia Ethical Committee (Approval Project Code: FF-287-2009).

### 4.3. Honey Treatment Protocol

HDFs were treated with 6 mg/mL of sterilized Gelam honey (w/v) with 24 h incubation. The concentration of Gelam honey was selected based on previous cytotoxicity study [15]. There were six different groups of HDFs viz. non-irradiated and non-honey treated HDFs (untreated control), irradiated HDFs and HDFs treated with Gelam honey alone. The other three groups were HDFs treated with Gelam honey pre-, during- and post-irradiation.

### 4.4. Exposure to Gamma-Irradiation

HDFs were exposed to gamma-rays at 1 Gray (Gy) using the ELDORADO 8 cobalt-60 source (Atomic Energy of Canada Ltd, Ontario, Canada) at the Secondary Standard Dosimetry Laboratory (SSDL, Malaysian Nuclear Agency). For 1 Gy of gamma-rays exposure, the dosage rate was calculated to be 0.25 Gy/min on the day the cells were irradiated. The cell culture flask was placed in the radiation chamber and the distance between the radiation source and the cell culture flask was 80 cm.

### 4.5. Primer Design

Primers for human *SOD1*, *SOD2*, *CAT*, *GPx1* and housekeeping gene (*GADPH*) were designed from listed NIH GenBank database using Primer 3 software and blasted against GenBank database sequences. The sequence for each primer is shown in Table 2.

### 4.6. RNA Extraction

Total RNA was extracted from HDFs using TRI Reagent (Molecular Research Center, Cincinnati, OH, USA). Polyacryl Carrier (Molecular Research Center) was added to precipitate the RNA before centrifuging to collect the RNA pellet. The RNA pellet was then washed with 75% ethanol and allowed to dry before adding RNase and DNase free distilled water to dissolve the pellet. All total RNA extracts were kept at −80 °C prior to use.

### 4.7. Quantitative Real-Time RT-PCR

Quantitative RT-PCR reaction was carried out using iScript One-Step RT-PCR Kit with SYBR Green (Bio-Rad, Hercules, CA, USA). Master mixes of RNA extract, nuclease-free H_2_O, 2X SYBR Green and reverse transcript (RT) solution were aliquotted into each reaction tube which contained forward and reverse primers. Reactions were conducted using iQ5 Bio-Rad iCycler with the following reaction profile; cDNA synthesis for 20 min at 50 °C, reverse transcriptase inactivation for 4 min at 95 °C and 38 cycles of PCR amplification of 10 sec at 95 °C and 30 sec at 61 °C. Melt curves were analysed at 95 °C for 1 min.

### 4.8. Protein Extraction

HDFs were harvested and centrifuged to collect the cell pellet. The pellet was resuspended in cold PBS (50 mM, pH 7.0) and incubated in ice for 10 min before centrifuged. Lysis buffer [complete mini EDTA-free (Roche, Indianapolis, USA) in RIPA buffer (Sigma, St. Louis, MO, USA) was added followed by incubation at 4 °C for 30 min. Suspension was then centrifuged to collect the supernatant which contains the enzyme extract. Protein concentration was determined by Bradford assay using bovine serum albumin as a standard protein. The total protein was expressed in mg/mL.

### 4.9. Enzyme Extraction

Approximately 1 × 10^6^ cells/mL of HDFs were collected and centrifuged to collect the cell pellet. The cell pellet was then resuspended in 50 mM PBS (pH 7.0) followed by sonication to extract the enzymes. Enzyme extract was then centrifuged to collect the supernatant which was used in the subsequent antioxidant enzyme assays.

### 4.10. Superoxide Dismutase (SOD) Assay

SOD assay was carried out according to the method as described previously [31]. Freshly prepared substrate which contained L-methionine, NBT, 1% Triton-X and PBS, pH 7.8 (Sigma) was mixed with enzyme extract before incubation under 20 watt of light in a cupboard for 7 min. Absorbance was measured using UV/VIS spectrophotometer (Shimadzu, Kyoto, Japan) at 560 nm wavelength. SOD specific activity/mg protein was expressed in mU/mg protein.

### 4.11. Catalase (CAT) Assay

Catalase activity was measured according to the method described previously [32]. Enzyme extract was pipetted into quartz cuvette before adding 30 mM H_2_O_2_, pH 7.0 (Merck, Darmstadt, Germany). The mixture was kinetically measured for 30 s using UV/VIS spectrophotometer (Shimadzu) at 240 nm wavelength. CAT specific activity was expressed in mU/mg protein.

### 4.12. Glutathione Peroxidase (GPx) Assay

The GPx assay was carried out using the method as described previously [33]. Freshly prepared substrate which contained GPx, reduced GSH, NADPH, sodium azide and PBS, pH 7.0 (Sigma) were mixed with enzyme extract before adding 0.0022 M hydrogen peroxide (H_2_O_2_) (Merck). The sample was then incubated for 5 min and kinetically measured using UV/VIS spectrophotometer (Shimadzu) at 340 nm wavelength. GPx specific activity was expressed in mU/mg protein.

### 4.13. Statistical Analysis

Experiments were carried out in duplicate with 3 independent cultures. Data are reported as means ± SD and comparison between groups were made by ANOVA. *p* < 0.05 was considered statistically significant.

## 5. Conclusions

Gamma-irradiation decreased the expression of SOD, CAT and GPx in HDFs. Gelam honey modulates the expression of antioxidant enzymes in irradiated HDFs by up-regulating *SOD* and *CAT* genes and their enzyme specific activities. The increased in SOD and CAT expression may counterbalance the production of ROS by radiation. These findings indicated that Gelam honey can act as a radioprotectant agent by maintaining the antioxidant defense system.

## Figures and Tables

**Table 1:** The specific activities of superoxide dismutase (SOD), catalase (CAT) and glutathione peroxidase (GPx) in different HDFs treatment groups.

Group of Treatment	Enzyme specific activity (mU/mg protein)		
	SOD	CAT	GPx
Control	1020.85 + 133.18	0.45 + 0.09	26.22 + 3.49
Irradiated HDFs	487.45 + 123.75 ^a^	0.32 + 0.05 ^a^	12.64 + 2.51 ^a^
Honey treatment	759.83 + 98.74 ^a,b^	0.47 + 0.13 ^b^	16.59 + 3.38 ^a,b^
Pre-honey treatment	772.06 + 206.36 ^a,b^	0.42 + 0.07 ^b^	13.12 + 2.92 ^a^
Honey treatment during radiation	665.04 + 114.80 ^a,b^	0.35 + 0.12	8.74 + 1.40 ^a,b,c,d^
Post-honey treatment	591.84 + 90.99 ^a,c^	0.36 + 0.08	6.16 + 0.50 ^a,b,c,e^

Results are expressed as means + S.D. (n = 6). ^a^ Denotes *p* < 0.05 compared to untreated control, ^b^ *p* < 0.05 compared to irradiated HDFs, ^c^ *p* < 0.05 compared to honey treated-HDFs, ^d^ *p* < 0.05 compared to pre-honey treated-HDFs, ^e^ *p* < 0.05 compared to honey treated-HDFs during irradiation.

**Table 2:** Primer sequences for quantitative real-time RT-PCR.

Gene	Sequence (5'–3')	Size of PCR product (bp)
*GAPDH* (Forward)	TCCCTGAGCTGAACGGGAAG	217
*GAPDH* (Reverse)	GGAGGAGTGGGTGTCGCTGT	
*SOD1* (Forward)	GAAGGTGTGGGGAAGCATTA	174
*SOD1* (Reverse)	ACATTGCCCAAGTCTCCAAC	
*SOD2* (Forward)	CGTCACCGAGGAGAAGTACC	312
*SOD2* (Reverse)	CTGATTTGGACAAGCAGCAA	
*CAT* (Forward)	CGTGCTGAATGAGGAACAGA	119
*CAT*(Reverse)	AGTCAGGGTGGACCTCAGTG	
*GPx1* (Forward)	CCAAGCTCATCACCTGGTCT	127
*GPx1* (Reverse)	TCGATGTCAATGGTCTGGAA	
